# *Journal of Personalized Medicine*—Aims and Scope Update

**DOI:** 10.3390/jpm15090436

**Published:** 2025-09-11

**Authors:** Kenneth P. H. Pritzker

**Affiliations:** Department of Laboratory Medicine and Pathobiology, Department of Surgery, Temerty Faculty of Medicine, University of Toronto, 1 Queens Park Crescent, Toronto, ON M5S 3H2, Canada; kenpritzker@gmail.com

## 1. Introduction

**Figure 1:**
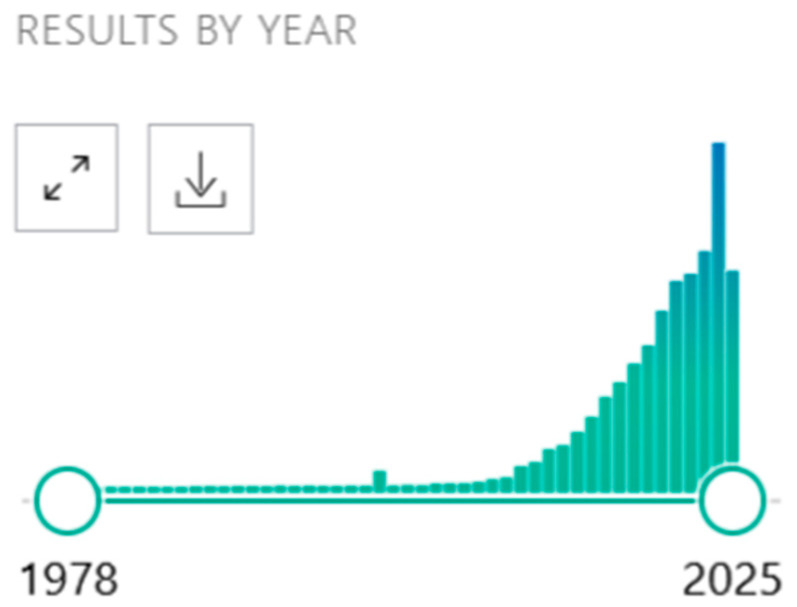
Personalized Medicine publications.

*Journal of Personalized Medicine* (*JPM*) [1,2] was established in 2011, with Prof. Dr. Urs A. Meyer as the founding Editor-in-Chief, as an international, open access journal dedicated to the emerging field of Personalized Medicine. As Prof. Dr. Meyers pointed out so clearly in *JPM*’s first issue, while the concept and practice has a history as long as medicine itself, in 2011, Personalized Medicine was just emerging as a formal investigative discipline [3]. Today, the field of Personalized Medicine is undergoing explosive growth in the number of publications; PubMed recorded just 556 papers in 2010, compared to 9330 in 2024 and more than 5800 in first 5 months of 2025 (Figure 1).

Contributing to Personalized Medicine’s development, the *Journal of Personalized Medicine* has grown and thrived under the visionary, enthusiastic leadership of its Editors-in-Chief (Table 1).

As part of the drive towards higher quality in scholarly journal publishing, citation-tracking organizations, such as Clairvate Web of Science and others, are encouraging journals to better define the scope incorporated in the Journal’s name and encourage submissions that best fit their stated scope.

Following current COPE guidelines [4], and under the guidance of the Editor-in-Chief, Prof. Dr. Kenneth P.H. Pritzker, with the collaboration and support of the Sections’ Editors-in-Chief, the journal has reviewed its scope and updated its aims so as to further clarify its role in the field. To help provide specific aims for *JPM* and focus on its renewed scope, a definition of “Personalized Medicine” is offered below.

Personalized Medicine, broadly understood, is the application of sciences, informatics, and humanities towards optimized disease prevention, diagnosis, and treatment for each individual human based on their unique genetic, acquired, and environmental state. It covers the processes of disease development—including disease risk factors, disease epidemiology, and prevention—disease mechanisms, disease diagnosis, disease treatment, and disease prognosis after treatment. Personalized Medicine is both a medical perspective and a process oriented towards truly individualized disease prevention, diagnosis, and treatment.

## 2. *JPM*—Updated Scope and Aims

### 2.1. Aims

*Journal of Personalized Medicine* (*JPM*; ISSN 2075-4426) is an international, open access journal aimed at bringing all aspects of Personalized Medicine together on one platform. *JPM* reports innovative preclinical and translational scientific research, technological advances, and novel clinical medicine applications that advance the study and implementation of Personalized Medicine, publishing research papers, reviews, editorials, communications, etc. Our aim is to encourage scientists to publish their experimental and theoretical results in as much detail as possible, with their full experimental details provided so that their results can be reproduced.

We provide a forum that brings together academic and clinical researchers, biotechnologists, diagnostic and pharmaceutical companies, health professionals, regulatory and ethical experts, and government and regulatory authorities. By deploying high-quality continuous improvement in all its processes, the Journal of Personalized Medicine seeks to be the most relevant and highest quality journal in its field, and aspires to be amongst the best journals across scholarly publishing.

### 2.2. Scope

For a comprehensive perspective comprising primary research, commentaries, and reviews related to Personalized Medicine, *JPM* aims to integrate expertise from the molecular and translational sciences, therapeutics, diagnostics, and medical care, as well as discussions of regulatory, social, ethical, and policy aspects. *JPM* particularly encourages submissions that focus on the following topics within its scope:

- Novel and emerging informatics applied to personalized medicine, including well-grounded artificial intelligence/machine learning-enabled methods.
- Diagnostic characteristics for disease subtypes, or evidence for groups of patients who are likely to respond to therapy vs. those who do not.
- Processes such as organ/tissue transplantation, where de-personalizing donor tissue results in broader positive outcomes for recipients.

Papers that may be considered as out of scope include the following:

- Methods papers that do not describe subpopulations where their approaches might be most or least effective.
- Reports and theoretical studies with broad implications only.

## 3. Updated Sections

Based on the updated aim and scope of the *Journal of Personalized Medicine*, its Sections have been restructured according to progress of disease development categories relevant to Personalized Medicine. The updated Sections will be as follows:

- Personalized Preventive Medicine;
- Diagnostics in Personalized Medicine;
- Personalized Therapy in Clinical Medicine;
- Personalized Medicine in Pharmacy;
- Personalized Medical Care;
- Mechanisms of Diseases;
- Omics/Informatics;
- Disease Biomarkers;
- Pharmacogenetics;
- Precision Oncology;
- General.

## Figures and Tables

**Table 1:** *Journal of Personalized Medicine* Editors-in-Chief.

Editor-in-Chief	Year
Prof. Dr. Urs A. Meyer	2011–2015
Prof. Dr. Stephen B. Liggett	2015–2021
Prof. Dr. David A. Rizzieri	2021–2024
Prof. Dr. Kenneth P.H. Pritzker	2024–now
